# Intriguing and Beautiful: *Adamacrocera adami* gen. et sp. nov. from the Upper Cretaceous Amber of Myanmar Represents a New Subfamily of Keroplatidae (Diptera: Bibionomorpha)

**DOI:** 10.3390/insects11090552

**Published:** 2020-08-19

**Authors:** Jan Ševčík, Wiesław Krzemiński, Kornelia Skibińska

**Affiliations:** 1 Department of Biology and Ecology, Faculty of Science, University of Ostrava, Chittussiho 10, 71000 Ostrava, Czech Republic; sevcikjan@hotmail.com; 2 Institute of Biology, Pedagogical University of Kraków, Podbrzezie 3, 31-054 Kraków, Poland; wieslawk4@gmail.com; 3 Institute of Systematics and Evolution of Animals, Polish Academy of Sciences, 31-016 Kraków, Poland

**Keywords:** fossil insects, Sciaroidea, inclusions, Mesozoic, taxonomy, new genus

## Abstract

**Simple Summary:**

The Upper Cretaceous amber of Myanmar (also known as Burmese amber) is almost 100 Mya old and represents invaluable source of information about the evolution of life in the late Mesozoic. Various groups of fossil flies (Diptera) belong to the most abundant insects found in these fossil resins indicating that it was the Mesozoic when the early evolution and radiation of Diptera took place. Here we describe a remarkable fossil fly which combines characters of various other related flies from different families (both extant and fossil) in a very unusual way. This new genus is tentatively placed in the family Keroplatidae (the so called predaceous fungus gnats) pending and stimulating further research into the evolution of lower Diptera.

**Abstract:**

A new fossil genus and species of Keroplatidae (Diptera, Bibionomorpha, Sciaroidea), *Adamacrocera adami* gen. et sp. nov., from the Upper Cretaceous Burmese amber is described and illustrated. Based on morphological evidence, it is placed in a new subfamily Adamacrocerinae subfam. nov. The new genus, as well as the subfamily, possesses the wing venation characteristic of the genera of some Sciaroidea incertae sedis, as well as that of the fossil families Archizelmiridae, Antefungivoridae and Mesosciophilidae, in combination with macrocerine-like habitus and male terminalia.

## 1. Introduction

The family Keroplatidae is one of the most diverse families of Sciaroidea in the dipteran infraorder Bibionomorpha [1], with nearly 1000 described extant species and about 50 fossil species, mostly from Baltic amber and other Tertiary deposits [2]. A real diversity of both the extant and fossil fauna of this family is still inadequately known, since new species and genera are being described almost every year [3,4,5,6,7]. The family is currently divided into six subfamilies: Arachnocampinae, Keroplatinae, Lygistorrhininae, Macrocerinae, Platyurinae and Sciarokeroplatinae [8].

The oldest described fossil species of Keroplatidae are *Hegalari minor* [9] and *H. antzinako* [9] (Macrocerinae, both from the Cretaceous Alava amber of Spain), followed by *Lebanognoriste* [10] (Lygistorrhininae, Lebanese Amber, ca. 130.0–125.5 mya), *Schlueterimyia cenomanica* [11] (Macrocerinae, Cretaceous amber of France) and *Burmacrocera petiolata* [12] (Macrocerinae, Cretaceous Burmese amber). The latter species was described by Cockerell [12] based on a single female and considered by him as closely allied to *Macrocera* [13]. Matile [14], however, placed the monotypic genus *Burmacrocera* (containing only *B. petiolata*) in the tribe Orfeliini of Keroplatinae. Actually, various specimens (both male and female) of *Burmacrocera* are relatively common in Burmese amber (personal collection of J. Ševčík), and there is no doubt that this genus belongs either to Macrocerinae or to a related archaic group, not to the much younger tribe Orfeliini, reliably known only since the Tertiary. However, the delimitation and systematic position of this Cretaceous genus is beyond the scope of this paper and will be the subject of a separate paper in preparation, covering also an undescribed extant Oriental genus near *Burmacrocera*.

The mid Cretaceous amber of northern Myanmar (Burmese amber) is considered as essential for understanding the origins and diversification of recent families of Sciaroidea [10]. Although this amber contains various taxa of Keroplatidae and related families [8], the vast majority of them still remain undescribed or unrecorded. Most of these Cretaceous taxa belong to the subfamily Macrocerinae or they are unplaceable to a subfamily.

The concept, synapomorphies and monophyly of the subfamily Macrocerinae are far from being clarified. The recent study by Mantič et al. [8] shows that this subfamily is most probably a rather heterogeneous group, monophyletic with high support only in a broader concept, including several genera regarded by these authors as Keroplatidae incertae sedis. The absence of a distinct morphological synapomorphy applies also to the entire family Keroplatidae in its current broad concept [8].

In this paper, we describe a new fossil genus of Keroplatidae, possessing a very unusual wing venation, leading us to the decision to establish a new subfamily for this genus. 

## 2. Materials and Methods 

The specimen was examined using a Nikon SMZ25 stereomicroscope equipped with a Nikon DS-Ri2 digital camera. Photomicrographs are focus stacks captured using this system and processed using NIS-Elements Imaging Software. Line drawings were produced by tracing photographs. The terminology principally follows that in Blagoderov and Ševčík [15] and Matile [14], while some terms of wing venation are after Krzemińska et al. [16]. The holotype comes from the Hukawng Valley in Kachin State, northern Myanmar, and it is deposited in the Silesian Museum, Opava, Czech Republic (SMOC). Burmese amber was dated by Cruickshank and Ko [17] to the middle–late Albian, but Grimaldi et al. [18] estimated the age of this resin to the Turonian–Cenomanian based on arthropod inclusions. Shi et al. [19], based on U-Pb dating of zircons from the volcaniclastic matrix of the amber, estimated the age of Burmese amber at 98.79 ± 0.62 Ma (earliest Cenomanian).

This published work and the nomenclatural acts it contains have been registered in ZooBank, the online registration system for the ICZN. The LSID for this publication is: LSIDurn: lsid: zoobank.org: pub: 610233EC-C775-43BC-8644-357EA9D45B95.

## 3. Results

### 3.1. Systematic Paleontology

Order Diptera Linnaeus, 1758 [20].

Infraorder Bibionomorpha Hennig, 1948 [21].

Superfamily Sciaroidea Billberg, 1820 [22].

Family Keroplatidae Rondani, 1856 [23].

### 3.2. Description of a New Fossil Material

Subfamily Adamacrocerinae subfam. nov.

LSID urn:lsid:zoobank.org:act:66D233EE-1BC3-4F00-9F3C-EAA05C1BA4D1

Type of genus *Adamacrocera* gen. nov.

Type of species *Adamacrocera adami* sp. nov.

Genera included *Adamacrocera* gen. nov.

**Figure 1:**
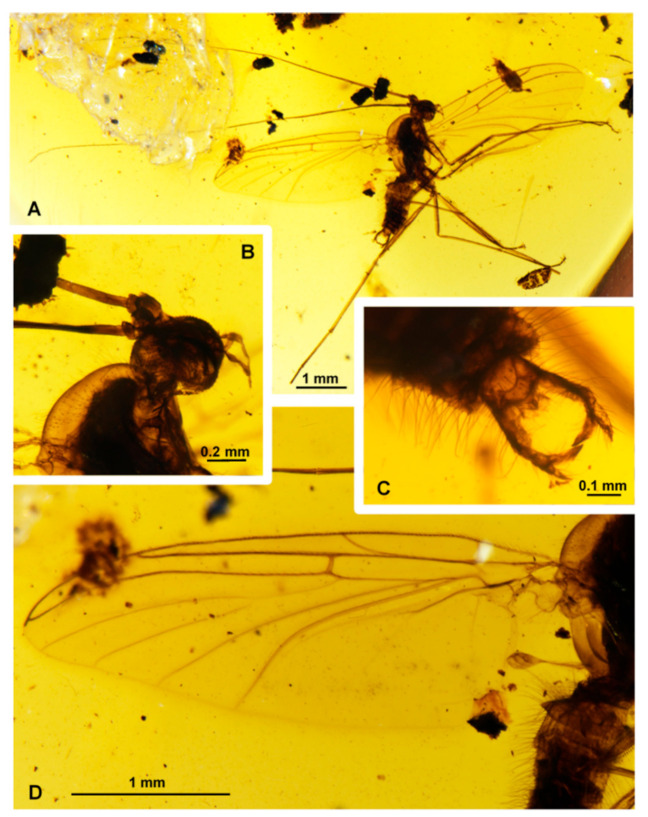
*Adamacrocera adami* gen. et sp. nov. Photos of the holotype [No. 426/2019]. (**A**) Whole specimen; (**B**) head; (**C**) male terminalia; (**D**) wing.

**Figure 2:**
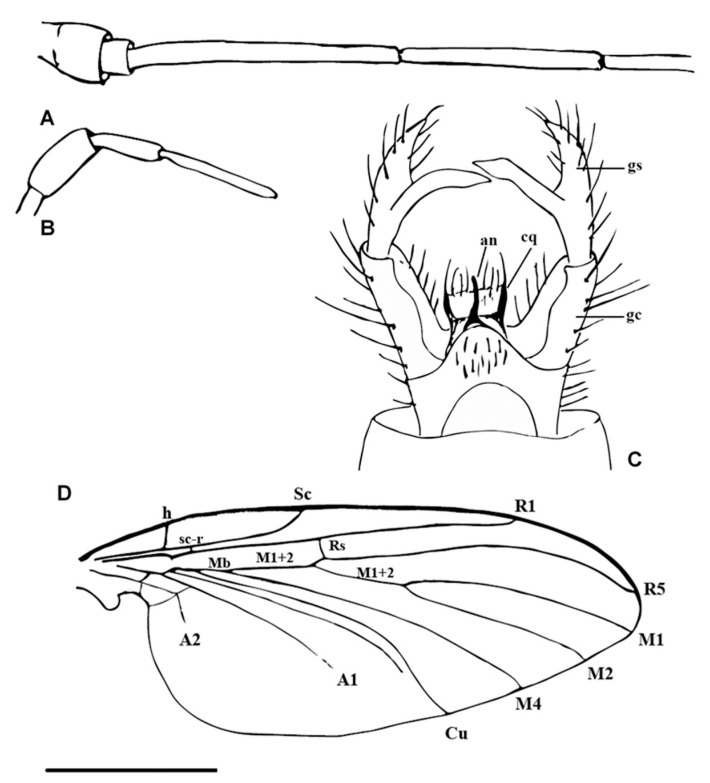
Line drawings of *Adamacrocera adami* gen. et spec. nov. (**A**) Antennae; (**B**) palpi; (**C**) male terminalia; (**D**) wing, scale: 1 mm. Abbreviations: gs—gonostylus; an—anus; cq—cerque; gc—gonocoxite; Sc—subcostal vein; sc-r—subcostal radial crossvein; h—humeral cross-vein; Rs—radial sector; R1—anterior branch of radius; R5—third branch of radius; M_1 + 2_—stem of media; M_1_—first branch of media; M_2_—second branch of media; M4—fourth branch of media; Cu—cubital vein; A_1_—first branch of anal vein; A_2_—second branch of anal vein.

Differential diagnosis. Medium-sized fungus gnats, superficially resembling Macrocerinae. Male antennae were about twice as long as the wing (Figure 1A). Wing vein Sc ends in C almost at the level of Rs; R_4_ was absent; bases of M _1 + 2_ were very long and nearly parallel with veins m-cu and Cub; r-m fusion was absent, A1 was short, not reaching the wing margin. The posterior part of the mediotergite (postphragma) was reduced (Figure 1). Male terminalia with gonostylus were as long as gonocoxite, deeply bifid in the posterior half, with both the branches of similar length (Figure 1C and Figure 2C).

*Adamacrocera* gen. nov.

LSID urn:lsid:zoobank.org:act:7D7E6F72-F5A6-4E25-953D-BE762B7E6FEA

Type of species: *Adamacrocera adami* gen. et sp. nov., by monotypy and present designation.

Diagnosis: the same as for the subfamily.

Etymology: the new genus (as well as the type species) was named after Adam Ševčík, a son of the senior author.

*Adamacrocera adami* sp. nov.

(Figure 1 and Figure 2)

LSID urn:lsid:zoobank.org:act:C10903B4-96E6-4293-89FB-C7840E21F0FA

Diagnosis: the same as for the genus and subfamily.

Description: body 2.20 mm long, wing length 3.42 mm.

Male antennae: 2.0 times as long as the wing and about 2.5 times as long as the body; scapus was short and very broad; pedicel was very short, tubular, much narrower than the scapus and only slightly wider than the first flagellomere; flagellomeres were bare, long and tubular (Figure 1A and Figure 2A).

Palpi with four visible palpomeres, the second palpomere was broader than the others; the apical segment was twice as long as the penultimate one (Figure 1B and Figure 2B).

Wings: 2.5 times as long as wide. Membrane hyaline was without macrotrichia and without visible markings. C ends slightly beyond the apex of R_5_; Sc terminates in C, almost at the level of Rs; sc-r ends before half of the Sc length and about three times its length after h; R_4_ was absent; r-m fusion was absent; Rs was short, oblique or transverse; Mb was absent; bases of M_1+2_ were very long (equal in length to M_1+2_) and cross vein m-cu and Cub were nearly parallel; A_1_ in the basal half was strongly sclerotized, and its distal part was very delicate and poorly visible, not reaching the wing margin; A_2_ was very short (Figure 1D and Figure 2D).

The thorax was about as high as long. The scutum was covered entirely with relatively long and thin hairs. The mediotergite was bare, evenly rounded and without distinct postphragma; the laterotergite was bare; the haltere was longer than the first abdominal segment (Figure 1A,D).

Legs with two tibial spurs on mid and hind leg: the inner one was about half the size of the outer one, fore tibia with a single spur, covered with regular, short and robust setae; tarsal claws, pulvilli and empodia were present but poorly visible. Length of legs in mm: front coxa/0.66, femur/1.01, tibia/1.03, tarsus 1/0.70, 2/0.22, 3/0.15, 4/0.07, 5/0.1; mid coxa/0.44, femur/0.97, tibia/1.11, tarsus 1/0.67, 2/0.25, 3/0.17, 4/0.04, 5/0.13; hind coxa/0.52, femur/1.56, tibia/1.69, tarsus 1/0.87, 2/0.42 3–5 absent (Figure 1A).

The abdomen was densely covered with long hairs. The hairs are about half as long as the breadth of the abdomen (Figure 1C,D).

Male terminalia: the gonocoxite was straight and relatively narrow; the gonostylus was as long as the gonocoxite, deeply bifid in the posterior half, with both the branches of similar length; tergite 9 was relatively short, convex in the posterior part and about twice as broad as long; cerci were apically pointed; hypoproct was dark and pointed. Aedeagus and the associated internal structures were not visible (Figure 1C and Figure 2C).

Material examined: Holotype (male), No. 426/2019; Burmese amber (the earliest Cenomanian, 98.79 ± 0.62 Ma), deposited in the Silesian Museum, Opava, Czech Republic (SMOC). 

Female unknown.

## 4. Discussions

Some of the body characters present in *Adamacrocera* gen. nov. are typical for Macrocerinae (Keroplatidae), especially the very long antennae and general outline of the male terminalia (Figure 1). On the other hand, a distinct cerebral sclerite, present in most Macrocerinae, is apparently missing in *Adamacrocera* gen. nov., and the wing venation is utterly different from any described genus of Keroplatidae (Figure 1D and Figure 2D). This unique combination of characters would even allow the establishment a new family for this genus. However, we consider this as premature, pending a discovery of further similar fossil genera, to see the variation of the wing and other characters, and also pending clarification of the family assignment of 20 genera (both extant and fossil) of fungus gnats unplaced to a family—the so-called Sciaroidea incertae sedis (sensu Jaschhof [24])—see below. We consider it as the best solution now to tentatively place this peculiar genus to a new subfamily of Keroplatidae, rather than to leave it unplaced or to describe a new family for it. 

The male terminalia of *Adamacrocera* gen. nov. are distinct in having a relatively long and deeply branched gonostylus (Figure 1C and Figure 2C), resembling some of the more primitive genera of Macrocerinae (Keroplatidae), namely *Chiasmoneura* (De Meijere, 1913) [25] (subgenus *Prochiasmoneura* Matile, 1988 [26]) and *Srilankana* (Matile, 1990) [14] and also *Heteropterna* (Skuse, 1888) [27] from the subfamily Keroplatinae. The structure of the male terminalia thus represents one of the most important indications that the new genus belongs to Keroplatidae. In most other Sciaroidea, including those unplaced to a family, male terminalia are much more complex, usually with shorter and unbranched gonostyli, or they are otherwise different.

Another character supporting the inclusion of *Adamacrocera* gen. nov. in Keroplatidae is the reduction in postphragma, the posterior part of the mediotergite (Figure 1D). It is particularly well developed in many genera of Sciaroidea incertae sedis (see Jaschhof [28]) but apparently not in *Adamacrocera* gen. nov.; at least, it is not traceable in the holotype. This is a more general problem specific to the study of fossil specimens that several characters are not visible, making the inclusion of these taxa in a cladistics analysis difficult. Sometimes fossils were included into a dataset, e.g., by Vilkamaa and Hippa [29], but with numerous characters coded as unknown, which may eventually affect the results.

On the other hand, the wing venation of *Adamacrocera* gen. nov. is very different from all the recent taxa of Keroplatidae, except for Arachnocampinae, but resembles various other taxa of Sciaroidea. A similar pattern of wing venation such as in *Adamacrocera* gen. nov. can be seen, for example, in *Catotricha* (Edwards, 1938) [30], a primitive genus of Cecidomyiidae, and also in several genera of the Sciaroidea incertae sedis, e.g., in *Eratomyia* (Amorim and Rindal, 2007) [31], *Chiletricha* (Chandler, 2002) [32], *Freemanomyia* (Jaschhof 2004) [28] (=*Pterogymnus* (Freeman, 1951) [33]), *Heterotricha* (Loew, 1850) [34] and *Starkomyia* (Jaschhof, 2004) [28]. The latter group of genera, still unplaced to a family, represents a real phylogenetic puzzle, most probably crucial for the understanding of the evolution of Sciaroidea [24,32]. Amorim and Rindal [31] placed most of these genera in the family Rangomaramidae sensu lato. This was, however, criticized by Jaschhof [24] who suggested using Sciaroidea incertae sedis for all the previously unplaced genera in the *Heterotricha* group sensu (Chandler) [32] and keeping the strict concept of Rangomaramidae with only the type genus included. Recently, Hippa and Ševčík [35] suggested including these genera into the Upper Jurassic and Lower Cretaceous family Antefungivoridae (=Pleciomimidae), although most of the morphological characters, except of wing venation, are unknown in these Jurassic taxa. Similarly, Chandler [32] and Jaschhof [28] pointed out the resemblance of the wing venation of *Freemanomyia* with that of the Mesozoic family Mesosciophilidae. However, most authors [24,29,32] agree that these genera do not constitute a single monophyletic group. This view is also supported by recent molecular studies [1,8,36].

In Burmese amber, there is currently just one described genus formally assigned to the Sciaroidea incertae sedis, *Thereotricha* (Blagoderov and Grimaldi, 2004) [10], although *Docidiadia* (Blagoderov and Grimaldi, 2004) [10] should be placed there too, rather than to Diadocidiidae, considering the relatively different male terminalia from recent diadocidiids and some other differences on wings and antennae. Both the latter genera differ considerably from *Adamacrocera* gen. nov., mainly in wing venation and the structure of the male terminalia.

It is of particular interest that the wing venation of the new genus resembles that of the fossil family Archizelmiridae, especially the Lower Cretaceous genus *Zelmiarcha* (Grimaldi, Amorim and Blagoderov, 2003) [37], although the proportions of particular veins, especially r-m and m-cu, are rather different. Additionally, the wing venation of some genera of Antefungivoridae and Mesosciophilidae is remarkably similar to *Adamacrocera*; see Kovalev [38] and Blagoderov [39]. This indicates that such a wing venation may represent a ground plan within Sciaroidea, formerly present in various lineages and retained only in several recent genera of Sciaroidea incertae sedis. A possible phylogenetic position of the family Archizelmiridae was discussed by Grimaldi et al. [37] and Vilkamaa and Hippa [29]. The former study suggests the closest relationship of Archizelmiridae with the Sciaroidea incertae sedis genera, while the latter includes this family in a broad concept of Sciaridae. Possible relationships of some genera of Sciaroidea incertae sedis with those of Antefungivoridae and Mesosciophilidae were discussed by Chandler [32], Hippa and Ševčík [36] and Jaschhof [28]. 

The complete vein Sc reaching the costa in *Adamacrocera* gen. nov. is a more plesiomorphic condition than in all the genera of Sciaroidea incertae sedis except for *Freemanomyia* and *Starkomyia*, while the latter retains R_4_ which is absent in the new genus. The absence of that vein also differs from the ground plan of the Keroplatidae and of the Macrocerinae, while long antennae are probably not in the ground plan of the family Keroplatidae. It is thus possible that the antennal structure has developed independently to that in Macrocerinae, so is not in itself evidence of a relationship to Keroplatidae. If we do not take male terminalia, reduced postphragma and long antennae into the account, there is no indication in the wing of *Adamacrocera* gen. nov. that it should belong to Keroplatidae but rather to the Sciaroidea incertae sedis group of genera, unplaced to a family or some Jurassic genus. Such a discrepancy between wing venation and other morphological features can be particularly misleading if there is only a wing preserved in the fossil record, like in many taxa of Bibionomorpha known only as Jurassic and other compression fossils.

Thus, there remains an open question if such a pattern of wing venation like in *Adamacrocera* gen. nov. could have evolved multiple times in different clades, including Keroplatidae, or, on the contrary, if the long antennae, reduction in postphragma and the *Macrocerinae*-like male terminalia are just an offshoot within the Sciaroidea incertae sedis or some fossil family. Considering the fact that various genera of the Sciaroidea incertae sedis most probably do not constitute a monophyletic group [1,24,29,32], the former hypothesis of the independent origin of the similar wing venation appears more plausible. Jaschhof [40] asserts that “wing venation in the Sciaroidea provides a character set that is notorious for the extent of homoplasy involved“. Given the relative simplicity of wing characters, it is easy to imagine that they are prone to parallel evolution. Parallel evolution of some characters is well documented either in Keroplatidae [5,8,41] or in the other groups of Sciaroidea, e.g., the absence of vein R_4_ in several genera of Keroplatidae belonging to different subfamilies (Arachnocampinae, Macrocerinae, Keroplatinae) or in the family Mycetophilidae [36]. This vein may be absent even in some species within a single genus (e.g., in *Macrocera*).

In any case, the inclusion of *Adamacrocera* gen. nov. in Keroplatidae makes this family even more heterogeneous than it used to be, so that it is now even more difficult to find a distinct synapomorphy for this family. The family Keroplatidae in the broad concept can thus be considered as a catch-all taxon, containing such different genera (in terms of wing venation), such as *Arachnocampa* (Edwards, 1924) [42], *Paleoplatyura* (Meunier, 1899) [43] and *Adamacrocera* gen. nov. This issue clearly requires further study, representing a real challenge for the future.

## 5. Conclusions

The new fossil genus, described in this paper, represents one of the most peculiar and remarkable sciaroid taxa currently known, combining characters of macrocerines (Keroplatidae) as well as of several genera of the Sciaroidea incertae sedis group, unplaced to a family, and also of the fossil families Archizelmiridae, Antefungivoridae and Mesosciophilidae. Although the wing venation pattern suggests that *Adamacrocera* gen. nov. represents a separate evolutionary line, we tentatively place it in the new subfamily of the family Keroplatidae, Adamacrocerinae subfam. nov., pending new findings of well-preserved Cretaceous fossils and further research into the evolution of fungus gnats (Sciaroidea) and lower Diptera as a whole.
